# Supplementary material for: RUNX1/CEBPA Mutation in Acute Myeloid Leukemia Promotes Hypermethylation and Indicates for Demethylation Therapy
Source: Int J Mol Sci. 2022 Sep 27;23(19):11413. doi: 10.3390/ijms231911413 (PMC9569612; doi:10.3390/ijms231911413)
Supplement: Supplementary file 1 [file ijms-23-11413-s001.zip › ijms-1694668-supplementary.pdf]

Supplementary Material

Tumor_Sample_Barcode	Hugo_Symbol	Chromosome	Start_Position	End_Position	Variant_Classification	Variant_Type	Protein_Change	i_TumorVAF_WU	Possible effect:	Mutation group	Cytogenetic_abnormality
TCGA-AB-2808	CEBPA	19	33792368	33792369	In_Frame_Ins	INS	p.317_318insM	42.86	altered target binding	CEBPA	normal
TCGA-AB-2808	CEBPA	19	33792368	33792369	In_Frame_Ins	INS	p.317_318insM	42.86		CEBPA	normal
TCGA-AB-2808	NRAS	1	115258744	115258744	Missense_Mutation	SNP	p.G13D	16.85		CEBPA	normal
TCGA-AB-2839	CEBPA	19	33792294	33792294	Frame_Shift_Del	DEL	p.R343fs	30.77	altered target binding	CEBPA	normal
TCGA-AB-2839	CEBPA	19	33792294	33792294	Frame_Shift_Del	DEL	p.R343fs	30.77		CEBPA	normal
TCGA-AB-2839	DNMT3A	2	25468133	25468133	Nonsense_Mutation	SNP	p.Q515*	55.56		CEBPA	normal
TCGA-AB-2839	NPM1	5	170837543	170837544	Frame_Shift_Ins	INS	p.-287fs	NA		CEBPA	normal
TCGA-AB-2839	NRAS	1	115258744	115258744	Missense_Mutation	SNP	p.G13D	37.63		CEBPA	normal
TCGA-AB-2839	WT1	11	32417908	32417909	Frame_Shift_Ins	INS	p.A170fs	NA		CEBPA	normal
TCGA-AB-2845	CEBPA	19	33792390	33792391	In_Frame_Ins	INS	p.310_311TQ>TKQNPQ	NA	altered target binding	CEBPA	normal
TCGA-AB-2845	CEBPA	19	33793190	33793191	Frame_Shift_Del	DEL	p.A44fs	0		CEBPA	normal
TCGA-AB-2845	CEBPA	19	33793191	33793191	Missense_Mutation	SNP	p.A44P	0		CEBPA	normal
TCGA-AB-2845	CEBPA	19	33792390	33792391	In_Frame_Ins	INS	p.310_311TQ>TKQNPQ	NA		CEBPA	normal
TCGA-AB-2845	CEBPA	19	33793190	33793191	Frame_Shift_Del	DEL	p.A44fs	0		CEBPA	normal
TCGA-AB-2845	CEBPA	19	33793191	33793191	Missense_Mutation	SNP	p.A44P	0		CEBPA	normal
TCGA-AB-2863	CEBPA	19	33792368	33792370	In_Frame_Del	DEL	p.T318del	25.93	altered target binding	CEBPA	8
TCGA-AB-2863	CEBPA	19	33792368	33792370	In_Frame_Del	DEL	p.T318del	25.93		CEBPA	8
TCGA-AB-2863	DNMT3A	2	25457242	25457242	Missense_Mutation	SNP	p.R882H	47.34		CEBPA	8
TCGA-AB-2874	CEBPA	19	33792423	33792423	Missense_Mutation	SNP	p.R300C	41.96	altered target binding	CEBPA	del(7q) / 7q-
TCGA-AB-2874	CEBPA	19	33792423	33792423	Missense_Mutation	SNP	p.R300C	41.96		CEBPA	del(7q) / 7q-
TCGA-AB-2874	WT1	11	32413566	32413566	Missense_Mutation	SNP	p.R250W	26.9		CEBPA	del(7q) / 7q-
TCGA-AB-2880	CEBPA	19	33792991	33792992	Frame_Shift_Ins	INS	p.G110fs	0		CEBPA	normal
TCGA-AB-2880	CEBPA	19	33792991	33792992	Frame_Shift_Ins	INS	p.G110fs	0		CEBPA	normal
TCGA-AB-2880	FLT3	13	28608251	28608252	In_Frame_Ins	INS	p.601_602insREYEYDL	NA		CEBPA	normal
TCGA-AB-2900	CEBPA	19	33792464	33792465	Frame_Shift_Ins	INS	p.R286fs	32.61	altered target binding	CEBPA	normal

TCGA-AB-2900	CEBPA	19	33792464	33792465	Frame_Shift_Ins	INS	p.R286fs	32.61		CEBPA	normal
TCGA-AB-2900	FLT3	13	28592642	28592642	Missense_Mutation	SNP	p.D835Y	37.5		CEBPA	normal
TCGA-AB-2900	NPM1	5	170837547	170837548	Frame_Shift_Ins	INS	p.WQ288fs	NA		CEBPA	normal
TCGA-AB-2940	CEBPA	19	33792395	33792396	In_Frame_Ins	INS	p.308_309insV	42.11	altered target binding	CEBPA	normal
TCGA-AB-2940	CEBPA	19	33793253	33793253	Frame_Shift_Del	DEL	p.P23fs	0		CEBPA	normal
TCGA-AB-2940	CEBPA	19	33792395	33792396	In_Frame_Ins	INS	p.308_309insV	42.11		CEBPA	normal
TCGA-AB-2940	CEBPA	19	33793253	33793253	Frame_Shift_Del	DEL	p.P23fs	0		CEBPA	normal
TCGA-AB-2952	CEBPA	19	33792895	33792896	Frame_Shift_Del	DEL	p.R142fs	0		CEBPA	complex - >= 3 distinct abnormalities
TCGA-AB-2952	CEBPA	19	33792895	33792896	Frame_Shift_Del	DEL	p.R142fs	0		CEBPA	complex - >= 3 distinct abnormalities
TCGA-AB-2952	NRAS	1	115258747	115258747	Missense_Mutation	SNP	p.G12D	11.55		CEBPA	complex - >= 3 distinct abnormalities
TCGA-AB-2955	CEBPA	19	33792404	33792405	In_Frame_Ins	INS	p.305_306insQ	50	altered target binding	CEBPA	normal
TCGA-AB-2955	CEBPA	19	33793184	33793184	Frame_Shift_Del	DEL	p.P46fs	0		CEBPA	normal
TCGA-AB-2955	CEBPA	19	33792404	33792405	In_Frame_Ins	INS	p.305_306insQ	50		CEBPA	normal
TCGA-AB-2955	CEBPA	19	33793184	33793184	Frame_Shift_Del	DEL	p.P46fs	0		CEBPA	normal
TCGA-AB-2955	DNMT3A	2	25457242	25457242	Missense_Mutation	SNP	p.R882H	40		CEBPA	normal
TCGA-AB-2979	CEBPA	19	33792395	33792400	In_Frame_Del	DEL	p.307_309NVE>K	NA	altered target binding	CEBPA	normal
TCGA-AB-2979	CEBPA	19	33793119	33793120	Frame_Shift_Del	DEL	p.I68fs	42.03		CEBPA	normal
TCGA-AB-2979	CEBPA	19	33792395	33792400	In_Frame_Del	DEL	p.307_309NVE>K	NA		CEBPA	normal
TCGA-AB-2979	CEBPA	19	33793119	33793120	Frame_Shift_Del	DEL	p.I68fs	42.03		CEBPA	normal
TCGA-AB-3000	CEBPA	19	33792390	33792391	In_Frame_Ins	INS	p.310_311TQ>TWQ	40.16	altered target binding	CEBPA	normal
TCGA-AB-3000	CEBPA	19	33793146	33793146	Nonsense_Mutation	SNP	p.E59*	0		CEBPA	normal
TCGA-AB-3000	CEBPA	19	33792390	33792391	In_Frame_Ins	INS	p.310_311TQ>TWQ	40.16		CEBPA	normal
TCGA-AB-3000	CEBPA	19	33793146	33793146	Nonsense_Mutation	SNP	p.E59*	0		CEBPA	normal
TCGA-AB-3008	CEBPA	19	33792408	33792409	In_Frame_Ins	INS	p.304_305KQ>KLQ	25.61	altered target binding	CEBPA	normal
TCGA-AB-3008	CEBPA	19	33792408	33792409	In_Frame_Ins	INS	p.304_305KQ>KLQ	25.61		CEBPA	normal
TCGA-AB-2805	RUNX1	21	36231783	36231783	Nonsense_Mutation	SNP	p.R174*	39.69	strongly reduced DNA binding,	RUNX1	normal

									loss of interaction with other proteins		
TCGA-AB-2805	RUNX1	21	36252939	36252940	Frame_Shift_Ins	INS	p.S114fs	34.69		RUNX1	normal
TCGA-AB-2805	IDH2	15	90631934	90631934	Missense_Mutation	SNP	p.R140Q	42.68		RUNX1	normal
TCGA-AB-2807	RUNX1	21	36252856	36252861	In_Frame_Del	DEL	p.SG140del	NA	altered DNA binding	RUNX1	NA
TCGA-AB-2807	ASXL1	20	31022727	31022727	Frame_Shift_Del	DEL	p.G738fs	50.41		RUNX1	NA
TCGA-AB-2807	IDH2	15	90631934	90631934	Missense_Mutation	SNP	p.R140Q	45.81		RUNX1	NA
TCGA-AB-2821	RUNX1	21	36252994	36252995	Frame_Shift_Ins	INS	p.D96fs	29.9		RUNX1	normal
TCGA-AB-2821	ASXL1	20	31022712	31022712	Nonsense_Mutation	SNP	p.Q733*	28.95		RUNX1	normal
TCGA-AB-2821	IDH2	15	90631838	90631838	Missense_Mutation	SNP	p.R172K	11.11		RUNX1	normal
TCGA-AB-2821	U2AF1	21	44514777	44514777	Missense_Mutation	SNP	p.Q157P	43.55		RUNX1	normal
TCGA-AB-2850	RUNX1	21	36164595	36164617	Frame_Shift_Del	DEL	p.GGERSPPR393fs	NA		RUNX1	normal
TCGA-AB-2850	IDH2	15	90631934	90631934	Missense_Mutation	SNP	p.R140Q	38.14		RUNX1	normal
TCGA-AB-2850	STAG2	23	123224615	123224615	Splice_Site	SNP		47.37		RUNX1	normal
TCGA-AB-2865	RUNX1	21	36231792	36231792	Missense_Mutation	SNP	p.D171N	60.62	strongly reduced DNA binding	RUNX1	NA
TCGA-AB-2865	RUNX1	21	36252940	36252940	Missense_Mutation	SNP	p.S114L	25.88		RUNX1	NA
TCGA-AB-2865	DIS3	13	73345997	73345997	Missense_Mutation	SNP	p.R514K	40.49		RUNX1	NA
TCGA-AB-2865	TET2	4	106157938	106157938	Nonsense_Mutation	SNP	p.Q947*	38.98		RUNX1	NA
TCGA-AB-2865	TET2	4	106193801	106193802	Frame_Shift_Ins	INS	p.K1422fs	32.46		RUNX1	NA
TCGA-AB-2865	TET2	4	106193802	106193803	Frame_Shift_Ins	INS	p.K1422fs	0		RUNX1	NA
TCGA-AB-2890	RUNX1	21	36231783	36231783	Nonsense_Mutation	SNP	p.R174*	24	strongly reduced DNA binding, loss of interaction with other proteins	RUNX1	normal
TCGA-AB-2899	RUNX1	21	36252878	36252878	Missense_Mutation	SNP	p.R135G	66.67	strongly reduced DNA binding	RUNX1	normal
TCGA-AB-2899	TCEAL6	23	101396171	101396171	Missense_Mutation	SNP	p.E45K	42.64		RUNX1	normal
TCGA-AB-2907	RUNX1	21	36231877	36231877	Splice_Site	SNP		44.44		RUNX1	normal
TCGA-AB-2907	ASXL1	20	31024758	31024758	Nonsense_Mutation	SNP	p.R1415*	35.19		RUNX1	normal
TCGA-AB-2907	IDH2	15	90631934	90631934	Missense_Mutation	SNP	p.R140Q	30.99		RUNX1	normal

TCGA-AB-2907	TTN	2	179647547	179647547	Missense_Mutation	SNP	p.Y1029C	27.95		RUNX1	normal
TCGA-AB-2912	RUNX1	21	36252877	36252877	Missense_Mutation	SNP	p.R135K	37.93	altered DNA binding	RUNX1	8
TCGA-AB-2912	PHF6	23	133511712	133511713	Frame_Shift_Ins	INS	p.N23fs	64.58		RUNX1	8
TCGA-AB-2912	U2AF1	21	44524456	44524456	Missense_Mutation	SNP	p.S34F	36.05		RUNX1	8
TCGA-AB-2927	RUNX1	21	36231783	36231783	Nonsense_Mutation	SNP	p.R174*	33.33	strongly reduced DNA binding, loss of interaction with other proteins	RUNX1	normal
TCGA-AB-2927	RUNX1	21	36252876	36252876	Missense_Mutation	SNP	p.R135S	42.86	strongly reduced DNA binding	RUNX1	normal
TCGA-AB-2927	ASXL1	20	31022234	31022234	Splice_Site	SNP		33.9		RUNX1	normal
TCGA-AB-2927	DIS3	13	73337717	73337717	Missense_Mutation	SNP	p.M667V	37.04		RUNX1	normal
TCGA-AB-2927	TTN	2	179484779	179484779	Missense_Mutation	SNP	p.D13814E	32.04		RUNX1	normal
TCGA-AB-2933	RUNX1	21	36252878	36252878	Missense_Mutation	SNP	p.R135G	58.33	strongly reduced DNA binding	RUNX1	normal
TCGA-AB-2936	RUNX1	21	36171607	36171607	Nonsense_Mutation	SNP	p.R293*	26.83		RUNX1	normal
TCGA-AB-2936	IDH2	15	90631934	90631934	Missense_Mutation	SNP	p.R140Q	39.13		RUNX1	normal
TCGA-AB-2936	TCEAL6	23	101396081	101396081	Nonsense_Mutation	SNP	p.E75*	42.36		RUNX1	normal
TCGA-AB-2959	RUNX1	21	36231782	36231782	Missense_Mutation	SNP	p.R174Q	86.96	strongly reduced DNA binding, naturally occurs in FPDMM	RUNX1	normal
TCGA-AB-2959	IDH2	15	90631934	90631934	Missense_Mutation	SNP	p.R140Q	42.11		RUNX1	normal
TCGA-AB-2959	PHF6	23	133547852	133547852	Splice_Site	SNP		64.71		RUNX1	normal
TCGA-AB-2970	RUNX1	21	36259153	36259153	Missense_Mutation	SNP	p.P86L	90.41769042	strongly reduced DNA binding	RUNX1	normal
TCGA-AB-2970	WT1	11	32413611	32413611	Splice_Site	SNP		43.38		RUNX1	normal
TCGA-AB-2978	RUNX1	21	36231783	36231783	Nonsense_Mutation	SNP	p.R174*	46.81	strongly reduced DNA binding, loss of interaction with other proteins	RUNX1	normal

TCGA-AB-2978	STAG2	23	123220476	123220476	Nonsense_Mutation	SNP	p.R1045*	20.33		RUNX1	normal
TCGA-AB-2978	TET2	4	106183007	106183008	Splice_Site	INS		0		RUNX1	normal
TCGA-AB-2978	TET2	4	106196213	106196213	Nonsense_Mutation	SNP	p.R1516*	40.79		RUNX1	normal
TCGA-AB-3009	RUNX1	21	36252869	36252870	Frame_Shift_Ins	INS	p.G138fs	38.05668016	altered DNA binding	RUNX1	normal
TCGA-AB-3009	PHF6	23	133551224	133551224	Missense_Mutation	SNP	p.G287V	93.491124260355		RUNX1	normal
TCGA-AB-3009	TTN	2	179472757	179472758	In_Frame_Ins	INS	p.15945_15945I>SL	40.95		RUNX1	normal
TCGA-AB-3009	WT1	11	32417909	32417910	Frame_Shift_Ins	INS	p.-169fs	41.0788381742739		RUNX1	normal

Supplemental Table S1. Mutations type description in each of the studied patient

ChIP-qPCR

The qPCR was performed with 5x qPCRmix-HS SYBR master mix (Evrogen, Moscow) and primers, specific to the region surrounding CpG of interest.

OSBPL5-1F	GCGGGAGGACAGTTATTCCTGA
OSBPL5-1R	CTCGGGAGTCGTGCCAACTT
BIK2-1F	GGGCTCTGGCCTCCTAAATG
BIK2-1R	GCTCCCAGACCACAGTAAACA
BIK2-2F	AATGAGCCACTCGTGGGGTA
BIK2-2R	AGACCACAGTAAACACTGCG

Supplemental Table S2. Primers overlapping the CpG associated with OSBPL5 and BIK genes.

Cell types

OCI-AML5 and OCI-AML2 cell lines (DSMZ cat. No.:ACC 247 and ACC 99, correspondingly) were used to represent RUNX1-mutated and RUNX1-wild type AML respectively. AML cell lines with RUNX1 mutations OCI-AML5 (c.361_362insTGCTA(The Institute for Cancer Research, UK n.d.)) and Mono-Mac1 (c.320C>T (“MONOMAC1” n.d.)) were used in a treatment experiment to represent cells with RUNX1 mutation.

Supplementary figures

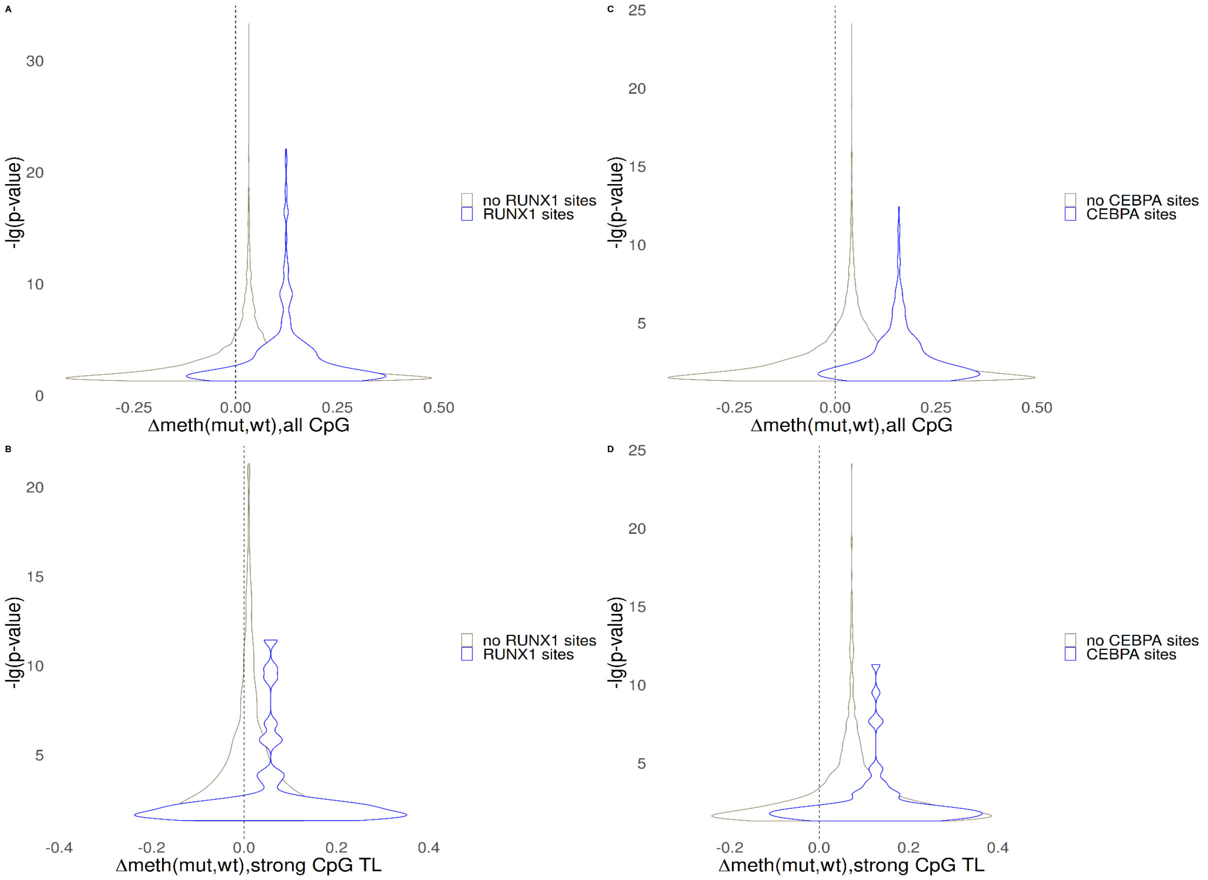

Supplemental Figure S1. Difference in the average levels of DNA methylation between AML patients with and without RUNX1/CEBPA mutation (*Δ*meth). Differentially methylated CpG in close proximity to TFBS and the rest of the differentially methylated CpGs are represented as purple and grey violins respectively.

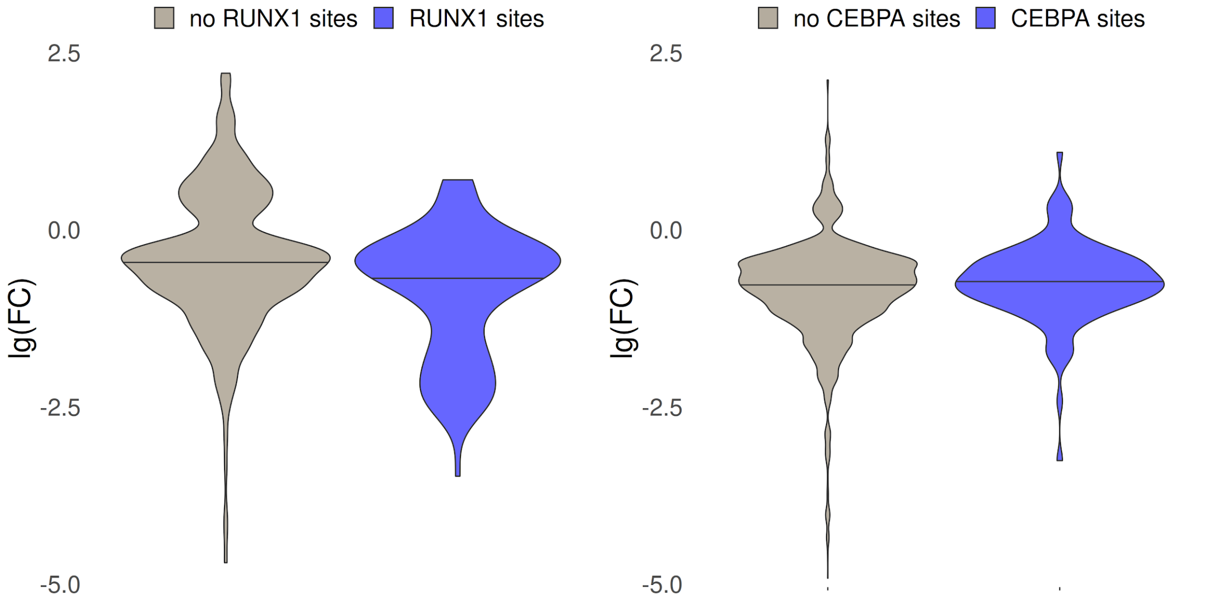

Supplemental Figure S2. Changes in expression of the genes regulated by RUNX1/CEBPA in AML patients with a mutation in a corresponding TF compared to patients without a mutation.

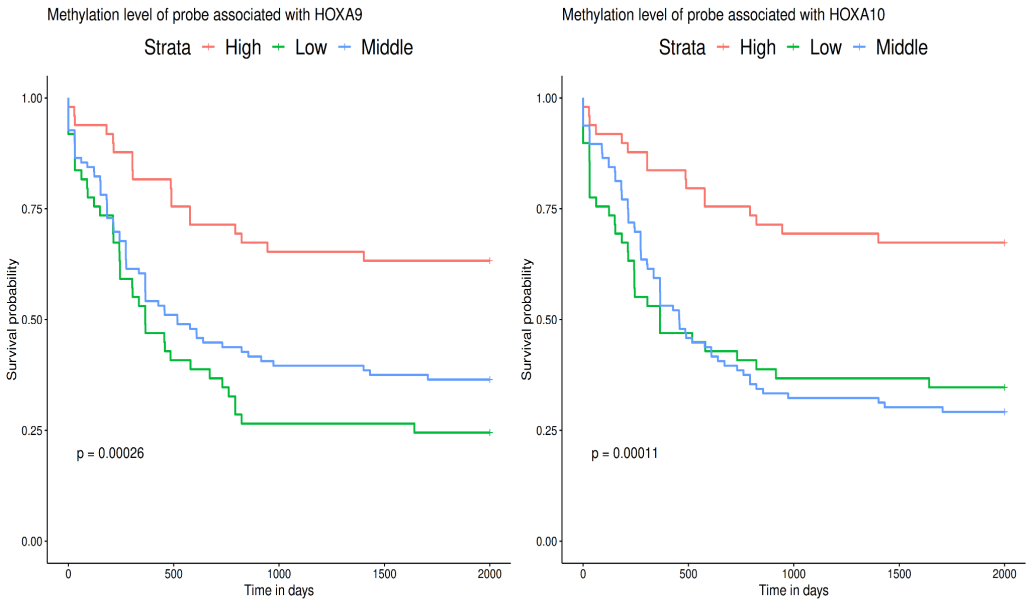

Supplemental Figure S3. Survival rates in patients with different levels of DNA methylation in HOXA9 and HOXA10 genes.

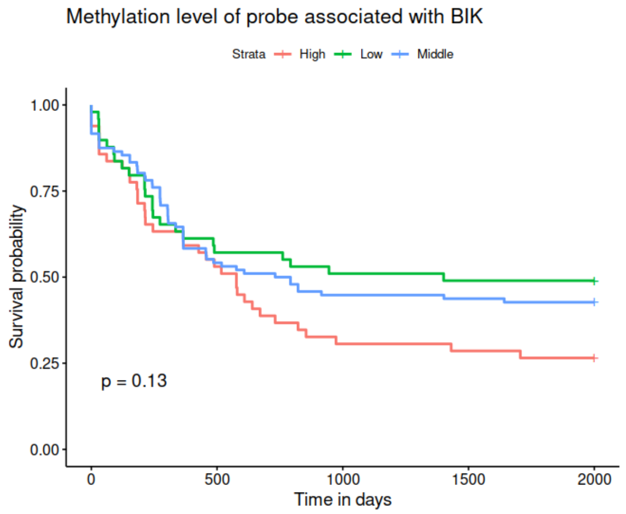

Supplemental Figure S4. Survival rates in patients with different levels of DNA methylation in BIK gene.

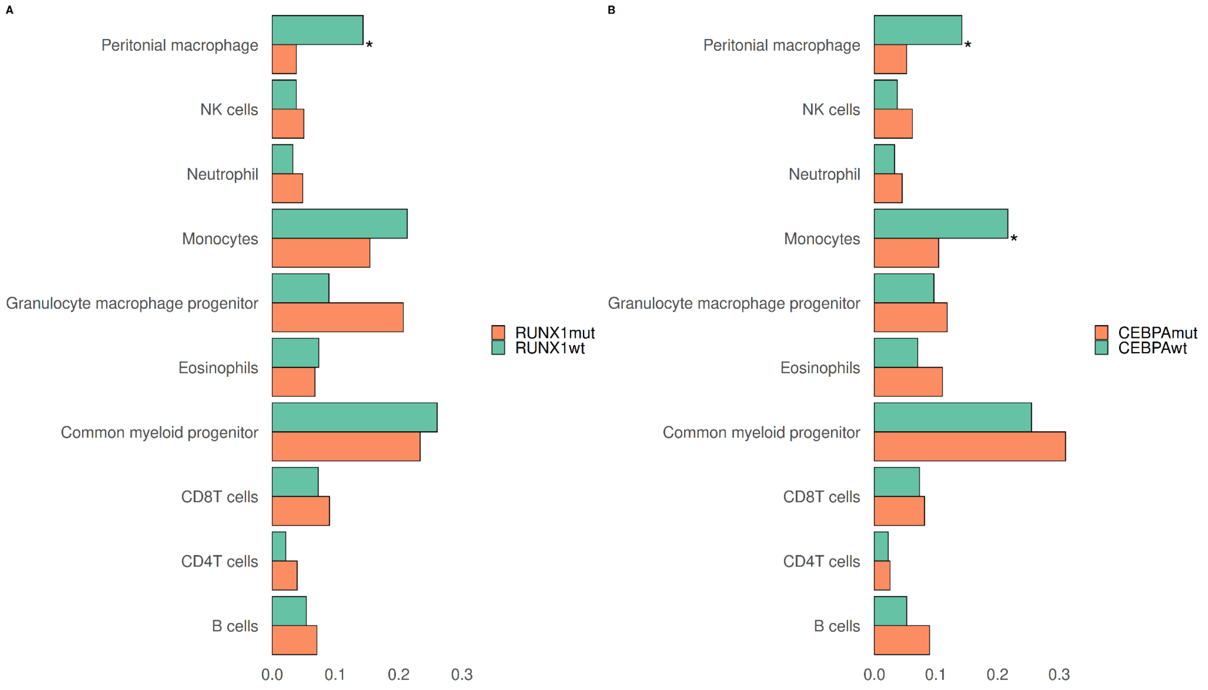

Supplemental Figure S5. (A,B) Fractions of the deconvoluted cell types in AML patients with and without RUNX1/CEBPA mutation. Significantly different cell types (p-value <0.05) are marked with star(*).

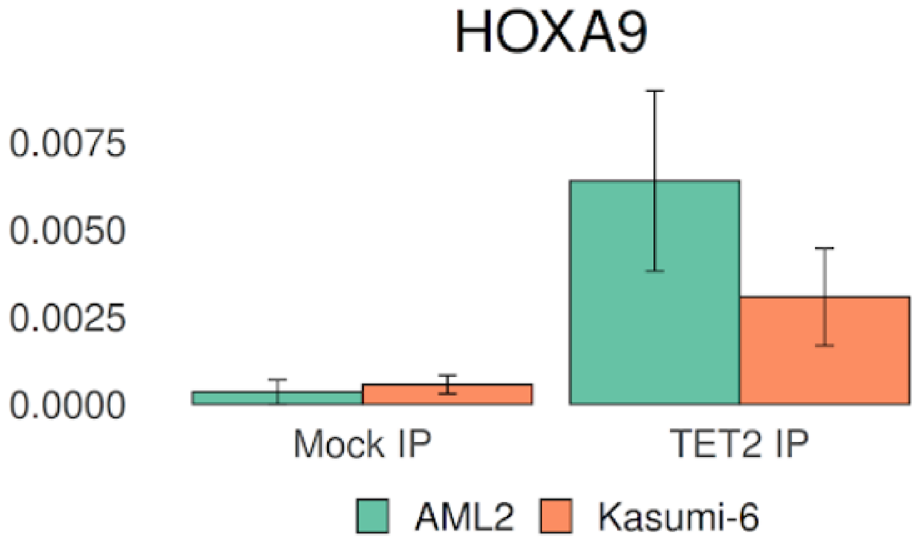

Supplementary Figure S6. TET2 binding in HOXA9 gene in OCI-AML2 cells (wt CEBPA) and in Kasumi-6 (mutated CEBPA). A mock IP was used as a background.

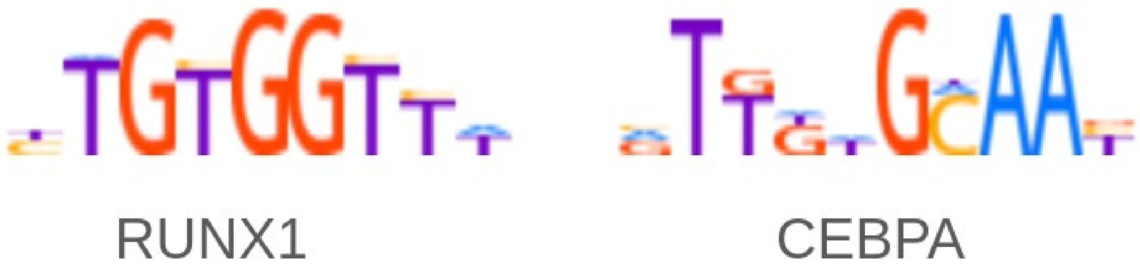

Supplementary Figure S7. RUNX1 and CEBPA motifs. (Kulakovskiy et al. 2018).

Gene name	CpG ID	Chromosome	Position	Gene expression, fold change RUNX1wt/ RUNX1mut	Gene expression, FDR	Delta DNA methylation (RUNX1mut-RUNX1wt)	DNA methylation, FDR
OSBPL5	cg14183540	11	3175007	2.594	0.000	0.377	0.000
BIK	cg05251389	22	43525330	4.796	0.000	0.320	0.000
C10orf91	cg15834395	10	134261413	2.164	0.000	0.271	0.004
C16orf93	cg01688936	16	30770794	2.120	0.000	0.265	0.027
VSTM1	cg01718139	19	54566838	2.330	0.009	0.255	0.004
ZBTB16	cg17518710	11	113954170	2.110	0.031	0.254	0.044
C20orf197	cg25724895	20	58630390	2.330	0.005	0.247	0.026
HOXB3	cg19710451	17	46654202	2.248	0.001	0.240	0.026
TNFRSF10C	cg18545076	8	22960177	2.236	0.016	0.225	0.015
LGALS3BP	cg04927537	17	76976091	7.399	0.000	0.217	0.004
KRT18	cg14212748	12	53343703	4.981	0.000	0.191	0.021
CACNA2D4	cg10665892	12	1921115	2.415	0.002	0.187	0.010
LGALS3BP	cg18749404	17	76975944	7.399	0.000	0.160	0.000

Supplemental Table S3. CpG positions and the corresponding genes that have the most dramatic change in methylation and expression in AML patients with the RUNX1 mutation (RUNX1mut) compared to AML patients with a wild type RUNX1 (RUNX1wt).

Gene name	CpG ID	Chromosome	Position	Gene expression, fold change CEBPAwt/ CEBPAmut	Gene expression, FDR	Delta DNA methylation (CEBPAmut-CEBPAwt)	DNA methylation, FDR
HOXA9	cg12600174	7	27205230	3.243	0.016	0.391	0.017
PTRF	cg27576485	17	40558063	3.267	0.041	0.349	0.018
HOXA10	cg21172377	7	27213893	3.781	0.002	0.296	0.019
SCHIP1	cg19605623	3	159579349	2.727	0.021	0.288	0.000
PTRF	cg11669285	17	40558061	3.267	0.041	0.274	0.022
GPR109B	cg25190513	12	123201362	2.286	0.004	0.273	0.010
GPR109B	cg15545247	12	123201372	2.286	0.004	0.270	0.007

Gene name	CpG ID	Chromosome	Position	Gene expression, fold change CEBPAwt/ CEBPAmut	Gene expression, FDR	Delta DNA methylation (CEBPAmut-CEBPAwt)	DNA methylation, FDR
HOXA9	cg12600174	7	27205230	3.243	0.016	0.391	0.017
PTRF	cg27576485	17	40558063	3.267	0.041	0.349	0.018
HOXA10	cg21172377	7	27213893	3.781	0.002	0.296	0.019
SCHIP1	cg19605623	3	159579349	2.727	0.021	0.288	0.000
PTRF	cg11669285	17	40558061	3.267	0.041	0.274	0.022
GPR109B	cg25190513	12	123201362	2.286	0.004	0.273	0.010
ECE1	cg13086983	1	21664810	2.063	0.025	0.212	0.040
PI4K2A	cg00997424	10	99400181	2.225	0.027	0.194	0.040
MFSD2A	cg01869896	1	40420299	2.291	0.031	0.192	0.001
TNS3	cg07488141	7	47560215	2.585	0.009	0.179	0.016
FSTL1	cg21790991	3	120137480	2.414	0.012	0.174	0.008
LOC283663	cg16075649	15	57595298	2.063	0.004	0.163	0.003

Supplemental Table S4. CpG positions and the corresponding genes that have the most dramatic change in methylation and expression in AML patients with the CEBPA mutation (CEBPAmut) compared to AML patients with a wild type CEBPA (CEBPAwt).

Kulakovskiy, Ivan V., Ilya E. Vorontsov, Ivan S. Yevshin, Ruslan N. Sharipov, Alla D. Fedorova, Eugene I. Rumynskiy, Yulia A. Medvedeva, et al. 2018. “HOCOMOCO: Towards a Complete Collection of Transcription Factor Binding Models for Human and Mouse via Large-Scale ChIP-Seq Analysis.” *Nucleic Acids Research* 46 (D1): D252–59.

“MONOMAC1.” n.d. Accessed April 12, 2021. https://depmap.org/portal/cell_line/ACH-001129?tab=mutation.

The Institute for Cancer Research, UK. n.d. “canSAR Black.” The Institute for Cancer Research, UK. Accessed April 12, 2021. https://cansarblack.icr.ac.uk/cell-line/OCI-AML-5/mutations.
